# A Comprehensive Risk Assessment and Stratification Model of Papillary Thyroid Carcinoma Based on the Autophagy-Related LncRNAs

**DOI:** 10.3389/fonc.2021.771556

**Published:** 2022-02-24

**Authors:** Yongrun Mu, Fuling Song, Kai Yuan, Zili Zhang, Yan Lu, Rongzhan Fu, Dongsheng Zhou

**Affiliations:** ^1^ Department of Head and Neck Surgery Department, Shandong Cancer Hospital Affiliated to Shandong First Medical University, Jinan, China; ^2^ Department of Cardiology, The First Affiliated Hospital of Shandong First Medical University & Shandong Provincial Qianfoshan Hospital, Jinan, China; ^3^ Department of Thyroid Surgery Department, The First Affiliated Hospital of Shandong First Medical University & Shandong Provincial Qianfoshan Hospital, Jinan, China; ^4^ The Fourth People’s Hospital of Jinan, The Third Affiliated Hospital of Shandong First Medical University, Jinan, China; ^5^ Shandong First Medical University, Jinan, China

**Keywords:** Autophagy, LncRNA, Papillary thyroid carcinoma, Prognosis, Sorafenib

## Abstract

**Background:**

Papillary thyroid carcinoma (PTC) is one of the most common malignant carcinomas in the endocrine system, and it has a growing incidence worldwide. Despite the development of diagnosis and treatment modalities for thyroid carcinoma, the outcome remains uncertain. Autophagy participates in the process of cancer invasion, malignancy, metastasis, and drug resistance. Emerging research has shown that long noncoding RNAs (lncRNAs) play an important role in the process of different types of cancers. However, the interaction between the process of autophagy and lncRNA and the value of autophagy-related lncRNA for risk assessment, prediction of drug sensitivity, and prognosis prediction in PTC patients remains unknown.

**Materials and Methods:**

We screened 1,283 autophagy-related lncRNAs and identified 144 lncRNAs with prognostic value in The Cancer Genome Atlas (TCGA) cohorts. Univariate and multivariate Cox regression analyses were used to establish the prognosis-related autophagy-related lncRNA risk classification consisting of 10 lncRNAs to indicate the level of risk, according to which the patients were grouped into high-risk group and low risk-group.

**Results:**

The high-risk group had dramatically worse overall survival compared with the low-risk group. Cox regression analysis was performed to confirm the independent prognostic value of the autophagy-related lncRNA risk stratification, and the time-dependent receiver operating characteristic curves of the risk stratification were 0.981 (1 year), 0.906 (3 years), and 0.963 (5 years). LncRNA CRNDE (LINC00180) is overexpressed in the tumor, and its high expression matched with poorer survival state. So, we chose it for further experiment. Finally, knockdown of the CRNDE in PTC increased the sensitivity to sorafenib.

**Conclusion:**

Collectively, we successfully established a novel risk stratification for PTC based on the expression profiles of autophagy-related lncRNAs.

## Introduction

Thyroid carcinoma is the most common endocrine malignant tumor, and 90% of this tumor type is made up of papillary carcinoma histologically (1). The improvement of thyroid-based diagnostic procedures, such as radiographic imaging and fine-needle aspiration, has contributed directly to the rapid increase of new discovered cases (2). Similarly, papillary carcinoma accounts for most of the increase (3). However, the other histological subtypes (anaplastic, medullary, and follicular) do not change markedly (4, 5). The sharp increase in mortality due to PTC has garnered increased concern from the public (3, 6, 7).

With the research on papillary carcinoma mainly focused on genetics, transcriptomics proteomics, and epigenetics, mechanistic knowledge is growing rapidly. Therefore, determining the correlation of the clinicopathological information with the genomic alternations and transcriptome has become a new area of research for many researchers (8).

The increasing risk of small thyroid nodes due to overdiagnosis alone remains controversial among clinical decision makers. Although the eighth edition of the American Joint Committee on Cancer–Union for International Cancer Control (AJCC–UICC) staging system describes that most patients at low risk have a lower mortality for differentiated thyroid cancer, there is a crucial discussion about initial therapeutic decision-making and the clinical management of those newly diagnosed (9), especially regarding the requirement for active surveillance or thyroid surgery (1).

Autophagy is a type II programmed cell death process, which directly controls physiological mechanisms by degradation of proteins and organelles to achieve homeostasis (10). It is an important pathway necessary to adjust to various stresses (11). Nevertheless, dysregulation of autophagy involved in multiple diseases, including cancer (12–14), results in both tumor suppression and oncogenesis at different stages of cancer development.

Long noncoding RNAs (lncRNAs) are a type of noncoding RNA whose length is more than 200 base pairs (15, 16). These play a role in diverse biological processes of cancer, such as tumor prognosis, immune system, and cell proliferation (17, 18).

There is still no proper risk stratification for guiding the decision maker to make the choice to perform surgery and for predicting the outcome following thyroidectomy. Here, we established a risk stratification based on the expression profiles of autophagy-related lncRNAs to predict the risk level of patients with PTC.

## Materials and Methods

### Thyroid Carcinoma Data Sets

We extracted the expression data and the matched clinical information of patients with papillary thyroid carcinoma (PTC) from The Cancer Genome Atlas (TCGA, https://cancergenome.nih.gov/). Then, we classified the RNAs into either protein-coding function or lncRNA using the Ensemble human genome browser (19). The expression data for all the messenger RNAs (mRNAs) and lncRNAs in the cohort were log2 transformed [log2(FPKM+1)] for downstream analyses. A total of 508 patients whose pathological subtype is PTC were included in the study, and those whose survival time was less than 30 days were excluded because their death is likely to be classified under surgery-related death.

### Cell Culture

PTC cell lines BCPAP, TPC‐1, and K1 were used in the following experiment. TPC-1 and K1 were cultured in Roswell Park Memorial Institute‐1640 medium (Gibco, USA) containing 10% fetal bovine serum (FBS) (Gibco, USA) and 1% antibiotics (P/S) (Gibco, NY, USA), and BCPAP was cultured in Dulbecco’s modified Eagle’s medium (DMEM) (Gibco, USA) containing 10% FBS (Gibco, USA) and 1% antibiotics. All samples were placed in an incubator (Thermo, USA) with 5% CO_2_ at 37°C. Cells were dissociated with 0.25% trypsin at a ratio of 1:3 when their density reached 80%.

### Cell Transfection

The small interfering RNAs (siRNA) were utilized for knocking down CRNDE, and scrambled siRNA (si‐NC) was used as the negative control. The sequences were shown in **Supplementary Table S1**. All of them were synthesized by RiboBio (Shanghai, China). Cell transfection was performed with Lipofectamine 2000 (Invitrogen, USA) according to the product instructions. After harvesting for 48 h, the efficiency of knocking down was identified using qRT-PCR.

### Cell Treatment and Proliferation Assay

Cell proliferation was measured using the Cell Counting Kit-8 (CCK-8) Assay (MedChemExpress, China) according to the manufacturer’s instructions. Briefly, PTC cells with and without transfection (4 × 10^3^ cells/well) were cultured in a 96-well plate in the presence or absence of sorafenib (MedChemExpress, China) (2 µM), and the cell viability was observed by measuring absorbance at 450 nm (24, 48, and 72 h) in a microplate reader after incubation with CCK-8 solution (10 µl) for 2 h.

### RNA Extraction and Quantitative Real-Time Polymerase Chain Reaction

TRIzol (Invitrogen, CA) was used to extract total RNA from PTC cells. Here, 1 ml of TRIzol reagent was added to a 3.5-cm dish and incubated for 10 min. Following this step, 0.2 ml of chloroform was added to the dish. All the contents of the dish were transferred into tubes and shaken vigorously for 16 s. After standing in ice for 10 min, the tubes were centrifuged at 12,000 rpm for 25 min at 4°C. Finally, the RNA was washed with 75% ethyl alcohol and dissolved in diethylpyrocarbonate (DEPC) water. Then, 1 µg extracted RNA retrieved in the above step was reverse-transcribed into cDNA in a 20-µl reaction volume by using the First Strand cDNA Synthesis Kit (Takara, Japan) according to the manufacturers’ instructions. The cDNA was used as a template for real-time PCR by using matched primers. The sequences used in quantitative PCR were shown in **Supplementary Table S1**. The length of amplified products was within 300 bp. The real-time PCR was performed with a 20-µl reaction system containing 2 µl cDNA, 2 µl of mix of primer, 4 µl dd H_2_O, and 10 µl SYBR Green Real-Time PCR Master Mix (Takara, Japan). The detailed reaction conditions were set by referring to the manufacturers’ instructions. Data were analyzed by using the ΔΔCt method, where the endogenous housekeeping gene *β-actin* was used as a quantity and quality control.

### Obtaining Autophagy-Associated Gene Sets and Identification of Autophagy-Related LncRNAs by Coexpression Analysis

A total of 232 autophagy genes were obtained from the Human Autophagy Database (HADb; http://www.autophagy.Lu), which provides a comprehensive and up-to-date list of human genes involved in autophagy and is shown in **Supplementary Table S2**. The Pearson correlation coefficient was calculated between the expression of autophagy genes and autophagy-related lncRNAs. Autophagy-related lncRNAs were selected based on the absolute value of the coefficient being >0.3 (|R|>0.3), and a P value <0.001 was considered significant. Detailed information on the correlation between lncRNAs and autophagy-related genes is shown in **Supplementary Table S3**.

### Construction of a Protein–LncRNA Interaction Network

To further explore the interaction between lncRNAs and autophagy genes, we established an lncRNA and mRNA network using the Search Tool for the Retrieval of Interacting Genes/Proteins (STRING). The software Cytoscape 3.8.1 was used to visualize the relationship between lncRNAs and mRNAs.

### Construction of the Prognostic Risk Stratification

Univariate and multivariate Cox regression analyses were performed to identify potential autophagy-related lncRNAs with prognostic value. The autophagy lncRNAs with a P value <0.01 were submitted to the next step to perform the multivariate Cox regression analysis. The risk stratification based on the expression of autophagy-related lncRNAs was established by using the candidate lncRNAs obtained from the multivariate Cox regression. The linear risk formula was RiskScore = (Coef1 × Expression Gene1) + (Coef2 × Expression Gene2) + (Coef3 × Expression Gene 3) + (Coef4 × Expression Gene4) + (Coef5 × Expression Gene5) +……+ (Coef Gene N × Expression Gene N).

Based on the expression profiles of autophagy-related lncRNAs, we conducted a principal component analysis (PCA) to investigate the difference between the high-risk group and low-risk group.

### Gene Set Enrichment Analysis

Gene set enrichment analysis (GSEA) was performed using GSEA software (Version 4.1.0). All operations were performed as previously described (19–22). The results of GSEA were visualized using an enrichment map. Each analysis of gene set permutation was calculated 1,000 times, and pathways enriched in specific phenotypes were sorted by normalized enrichment score (NES) and P value. Relevant gene sets were obtained from the gene set database of Kyoto Encyclopedia of Genes and Genomes (KEGG) pathways detailed in **Supplementary Figure S2**.

### Statistical Analysis

A Cox regression model was employed to construct the risk stratification. The hazard ratio (HR) and associated 95% confidence interval (CI) were calculated. From the analysis of two subgroups based on the median value of risk score, the overall survival (OS) times of the high-risk and low-risk subgroups were calculated and analyzed *via* Kaplan–Meier test and compared using the log-rank test. The time-dependent receiver operating characteristic (ROC) curve was used to evaluate the predictive accuracy of the autophagy-related lncRNAs.

## Results

### Identification of Prognostic Autophagy-Related LncRNAs in Papillary Thyroid Carcinoma

**Figure 1:**
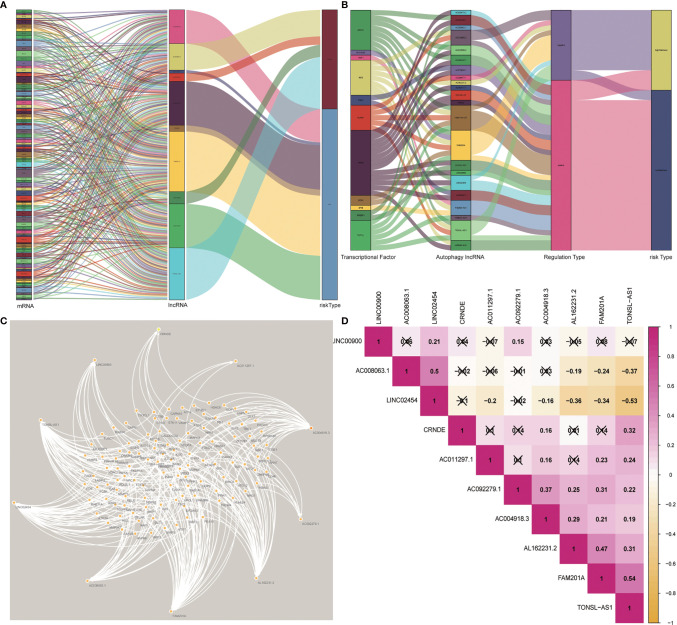
Construction of the regulation network of the long noncoding RNAs (lncRNAs) with prognostic value and autophagy genes in papillary thyroid carcinoma (PTC). **(A)** The coexpression network of the 10 autophagy-related lncRNAs–messenger RNAs (mRNAs) with prognostic value was constructed and visualized using Sankey diagram between prognostic risk-related lncRNAs, mRNAs, and risk types (risk or protective). **(B)** The coexpression network of autophagy-related lncRNAs and matched transcriptional factors was constructed and visualized using Sankey diagram. **(C)** The network of autophagy gene and autophagy-related lncRNA. **(D)** The correlation of expression levels of lncRNAs involved in the risk stratification.

By constructing the coexpression network of the 232 autophagy genes, a total of 1,283 autophagy-related lncRNAs were obtained (**Figures 1A–D**). Then, we performed Cox proportional hazards analysis to obtain 144 autophagy lncRNAs with significant prognostic value by analyzing the expression profile (**Figure 1**). There were 32 lncRNAs with low risk (HR <1) and 112 lncRNAs with high risk (HR >1) (**Supplementary Table S4**). Following this, multivariate Cox analysis was performed to further screen 10 lncRNAs from 144 autophagy lncRNAs with prognostic value, and they were AC008063.1, AC011297.1, FAM201A, AC092279.1, LINC00900, AL162231.2, CRNDE, TONSL-AS1, LINC02454, and AC004918.3 (**Figures 4A–J**) (**Supplementary Table S5**).

### Construction of a Prognostic Model

Then, these optional lncRNAs were submitted to the next step to construct the prognostic risk stratification of autophagy-related lncRNAs. We established a coexpression network of autophagy-related lncRNAs with prognostic value and mRNAs, as shown in (**Figures 1A, C**). And the formula is as follows:

Risk score = (AC008063.1*-1.740157523) + (AC011297.1*0.171231624) + (FAM201A*0.895715416) + (AC092279.1*-3.730086976) + (LINC00900*-2.38496601) + (AL162231.2*0.671649156) + (CRNDE*0.450612252) + (TONSL-AS1*-0.79310585) + (LINC02454*0.588168097) + (AC004918.3*2.412626944).

**Figure 2:**
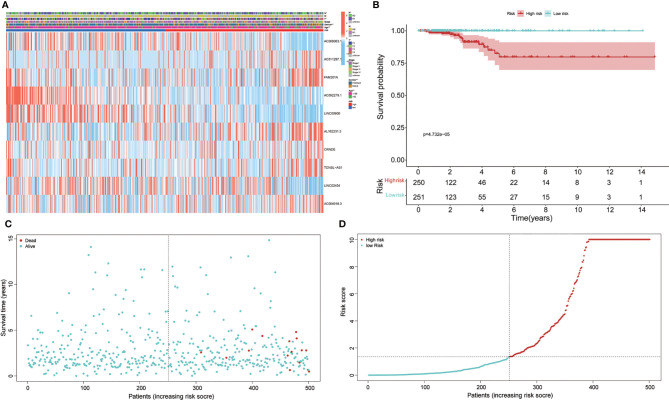
Prognosis and expression of risk genes in the two groups of papillary thyroid carcinoma (PTC) patients. **(A)** The heatmap displayed the expression levels of autophagy-related long noncoding RNAs (lncRNAs) in the high-risk and low-risk groups. **(B)** Kaplan–Meier survival analysis of the high-risk and low-risk groups based on the risk stratification. **(C)** The scatter plot based on the survival status of each patient. The different colors matched the status of survival and death. **(D)** The risk curve was drawn based on the risk score of each sample. The different colors matched the status of risk.

Based on the risk formula and median risk score, the PTC patients were divided into a high-risk group and a low-risk group (**Figure 2A**). Kaplan–Meier survival analysis showed that patients in the high-risk group had shorter OS than that in the low-risk group (**Figure 2B**), which suggested that the risk stratification had good prognostic prediction ability.

**Figure 3:**
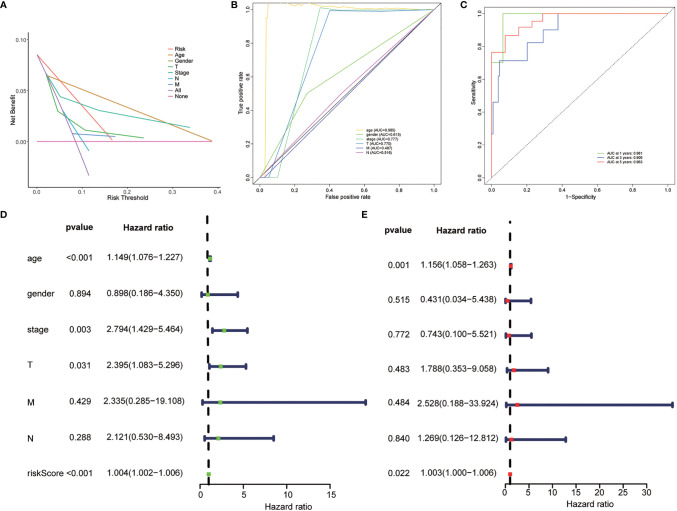
Evaluation of the clinical utility of the risk stratification. **(A)** DCA curve was drawn based on the risk stratification. **(B)** The area under the curve (AUC) value of clinical features according to the receiver operating characteristic (ROC) curves. Clinical features: Age, TNM stage, gender, T (tumor size), N (lymph node metastasis), and M (distant metastasis). **(C)** ROC curve of the risk stratification showing the prognostic performance of the autophagy-related long noncoding RNA (lncRNA) risk stratification. **(D)** The univariate Cox regression analysis of the risk model score and clinical features regarding prognostic value. **(E)** The multivariate Cox regression analysis of the risk model score and clinical features regarding prognostic value.

A scatter plot of survival status and risk curve were made in order to display the risk score and the corresponding survival status of PTC patients, the results of which showed that the higher risk score corresponded to higher mortality. At the same time, the heatmap was drawn based on these 10 autophagy-related lncRNAs in the PTC samples (**Figure 2A**). The survival curve (**Figures 2B–D**). The heatmaps displayed the expression of these elements in the high-risk group and low-risk group. Hence, these results indicate that all 10 autophagy lncRNAs have good prognostic value for PTC (**Figures 2**, **3**).

### Evaluation of the Risk Stratification Based on the 10 Autophagy-Related LncRNAs as an Independent Prognostic Factor for Papillary Cancer Patients

Univariate and multivariate Cox regression analyses were employed to determine whether the risk stratification based on autophagy-related lncRNAs was an independent prognostic factor of PTC. The HR and 95% CI of the risk score based on the risk stratification were 1.003014104–1.00616136988199 (P < 0.05) in the univariate Cox regression analysis and 1.004196324–1.00559955694039 (P < 0.05) in the multivariate Cox regression analysis (**Figures 3D, E**). The results showed that the risk stratification based on autophagy-related lncRNAs was a powerful significant prognostic factor of PTC, independent of clinicopathological characteristics such as tumor size, metastasis of lymph node and distant metastasis, sex, and TNM stage. The ROC curve was plotted to evaluate and estimate the predictive specificity and sensitivity of the risk stratification based on the autophagy-related lncRNAs (**Figure 8**). The time-dependent area under the curve (AUC) for 1, 3, and 5 years was 0.981, 0.901, and 0.963, respectively (**Figure 3B**). At the same time, the AUC of the other clinical characteristic including T, N, M, age, and TNM (stage) was calculated, and the AUC value of the risk score exceeded most of the others (**Figure 3C**). These results indicate that the risk stratification based on autophagy-related lncRNAs was an effective independent prognostic factor for PTC patients. Subgroup analysis was performed based on the risk stratification (**Figures 5C–N**). Besides, those patients both with high tumor mutation burden (TMB) levels and high risk exhibited a poorer survival state (**Figures 5A, B**). PCA results show that the model has an excellent ability to distinguish high-risk and low-risk patients (**Figures 6A–D**). DNA stemness score (DNAss) and RNA stemness score (RNAss) presented significant differences among the two subgroups (**Figures 6E, F**). Different risk types possessed different TIME statuses, Immunogenomics features (**Figure 7**).

**Figure 4:**
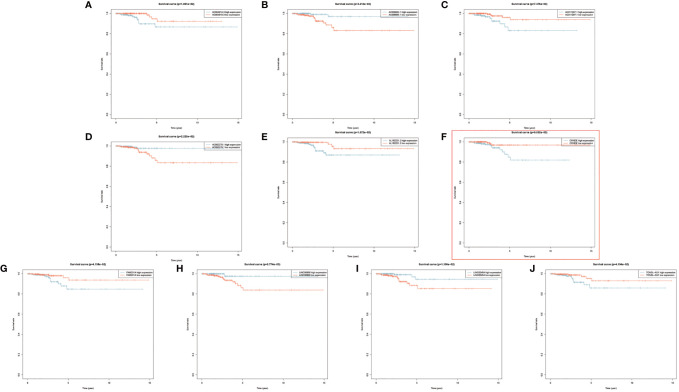
**(A–J)** Kaplan–Meier survival analyses of 10 prognostic risk-related autophagy-related long noncoding RNA (lncRNA).

**Figure 5:**
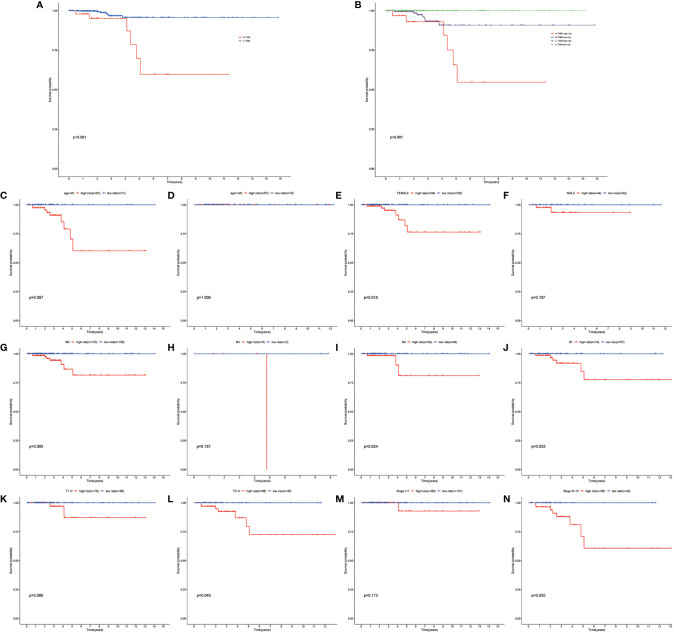
Performing the survival analysis combining TMB with risk stratification. **(A)** Kaplan–Meier survival analyses of high-TMB and low-TMB papillary thyroid carcinoma (PTC) group. **(B)** Kaplan–Meier survival analyses of high TMB combined with high risk, high TMB combined with low risk, low TMB combined with high-risk group, and low TMB combined with low-risk group. **(C–N)** Kaplan–Meier survival analyses of the autophagy-related long noncoding RNA (lncRNA) risk stratification in different subgroups.

**Figure 6:**
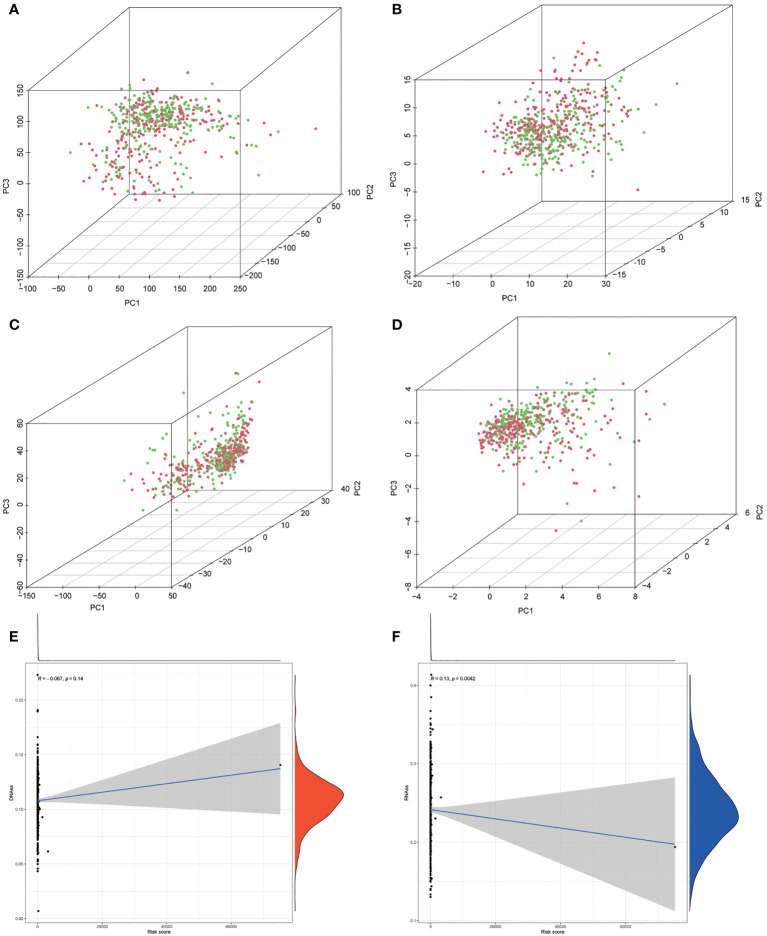
**(A–D)** Principal component analysis (PCA) between the low-risk and high-risk groups based on risk stratification. **(A)** The whole genome. **(B)** Autophagy-related encoding genes. **(C)** Autophagy-related long noncoding RNA (lncRNA). **(D)** The risk model of the 10 autophagy-related lncRNA expression profiles. **(E, F)** The correlation among risk stratification and tumor stem cell score (based on RNA expression and DNA methylation). **(E)** The correlation between the risk stratification and DNA stemness index. **(F)** The correlation between the risk stratification and RNA stemness index.

**Figure 7:**
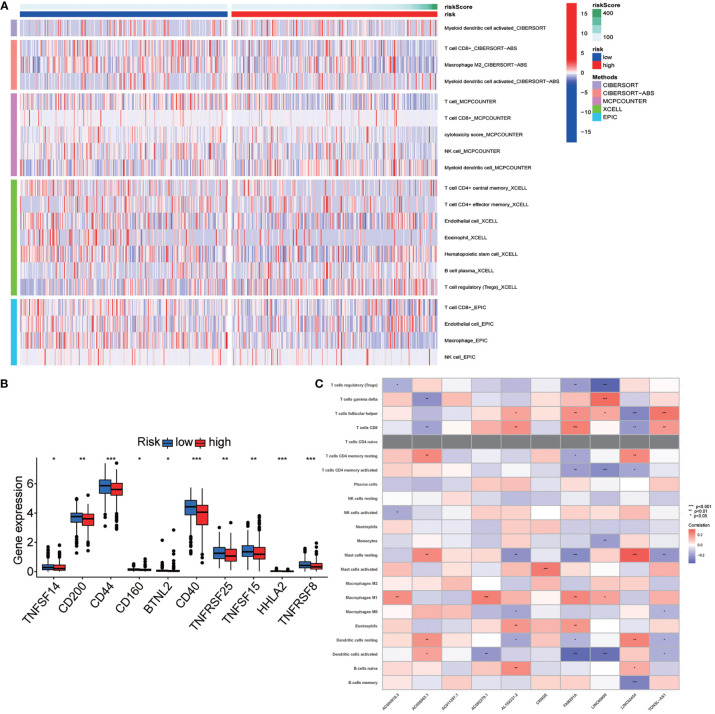
Exploration of the association of the risk stratification with tumor immunity. **(A)** Immune infiltration statuses among high-risk group and low-risk group through TIMER, CIBERSORT, XCELL, QUANTISEQ, MCPcounter, EPIC, and CIBERSORT database. **(B)** Differential expression checkpoint gene in papillary thyroid carcinoma (PTC) sample of high-risk and low-risk patients. **(C)** Correlation between the long noncoding RNA (lncRNA) involved in the risk stratification and immune cell infiltration. Symbols *, **, *** means that p-value <0.05, <0.01 and <0.001, respectively.

**Figure 8:**
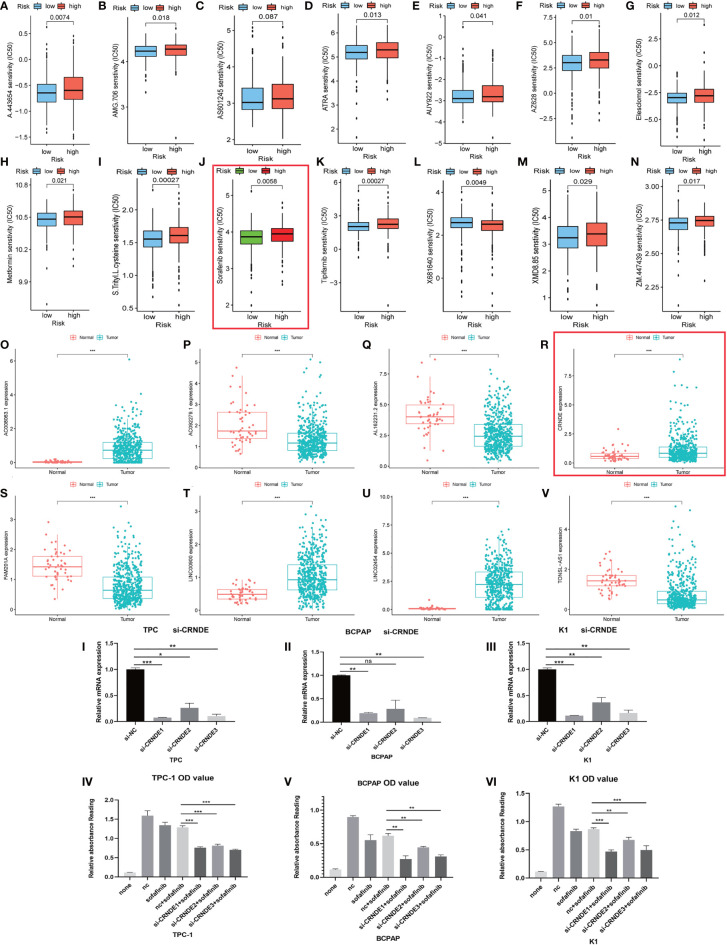
Evaluation and valuation of the prediction ability of sensitivity of risk stratification for common clinical chemotherapies and targeted therapies. **(A–N)** The risk stratification also can act as a powerful predictor of chemosensitivity for clinical chemotherapeutics. **(O–V)** The expression of 10 long noncoding RNAs (lncRNAs) in papillary thyroid carcinoma (PTC) and normal tissue. (IV–VI) Here, 2 µM sorafenib was added after knocking down CRNDE in three PTC cell lines. I-III: Efficiency of knocking down were confirmed with qRT-PCR. Symbols *, **, *** means that p-value <0.05, <0.01 and <0.001, respectively. The expanded form of ns means that no significance.

### Knocking Down CRNDE Significantly Improves the Sensitivity of Papillary Thyroid Carcinoma Cell Line for Sorafenib

Interestingly, most of the risk genes involved in the stratification system with higher expression in PTC samples matched better prognosis (**Figures 4F**, **8R**). After further validation, we found that those patients with higher CRNDE expression matched worse survival state.

We tried to evaluate the association between the risk stratification and the efficacy of chemotherapeutics based on the THCA cohort of TCGA project. Interestingly, the results showed that higher risk score based on the risk stratification was associated with higher half inhibitory concentration (IC50) of sorafenib (P < 0.01) (**Figure 8K**).

Next, we chose lncRNA CRNDE from the 10 autophagy lncRNAs and investigated if it could mediate cancer cell survival under sorafenib challenge. By directly knocking down CRNDE in the PTC cell line (Bacap-1, TPC-1, and K1), CCK-8 assay showed that knocking down CRNDE partially increased the effects of sorafenib-induced cell death in PTC cells with si-CRNDE transfection (**Figures 8I–III**). All those results suggested that CRNDE plays a positive role in tumorigenesis and may regulate the sorafenib sensitivity of PTC cells (**Figures 8IV–VI**).

## Discussion

Over the past year, the diagnostic rate of thyroid cancer has increased rapidly because of the popularization and widespread application of various imaging techniques. PTC lists on top of head and neck cancers. Currently, the comprehensive treatment strategy of combining surgery, thyroid hormone therapy, and internal radiotherapy is widely accepted. However, the definitive molecular mechanisms contributing to the malignant phenotype of PTC remain poorly explored. The high occurrence of thyroid cancer has been shown to have a close relationship with daily iodine intake and disorders of thyroid-stimulating hormone (TSH) levels, and a strong association was also proven that some PTC patients exposited to ionizing radiation (23).

Abnormal expression state of specific genes including tumor suppressor genes and oncogenes and epigenetics process such as methylation of promoters and acetylation of histone are involved in the heterogeneity of PTC. The common classical oncogenic alterations are found in the tumorigenesis of PTC, among which activating mutation in BRAF lists as first among common genetic alterations. BRAF V600E mutation has been verified as a powerful prognostic marker to evaluate the risk of PTC (24). The mutation of BRAF gene has a strong correlation with aggressive clinical characteristics such as metastasis of lymph node, extra thyroid diffusion, high recurrence rate, and resistance to radioactive iodine. Mutation of BRAF reduces the ability of cancer cells to take up iodine. The status of telomerase reverse transcriptase (TERT) mutation has been discovered to serve as an independent prognostic factor for PTC (25). NTRK, HRAS, KRAS, NRAS, RET, RET/PTC, and PAX8/PPARγ gene mutations were proven as oncogenic driver mutations in tumorigenesis of PTC (26–28). The unique oncogene duet of coexisting BRAF V600E and TERT promoter mutations is widely proven to be a robust genetic background promoting thyroid cancer aggressiveness (29–31). Systematic identification and analysis of these oncogenic alterations will help clinical decision makers better diagnose, predict the prognosis, and make the appropriate treatment decision.

Currently, the exploration of molecular biomarkers and development of relative risk stratification are of great interest in cancer research, including screening for new effective diagnostic biomarkers of early-stage cancer, establishing new risk stratifications to predict the OS of cancer patients and new drug research and development.

The discovery of thousands of noncoding RNAs has changed the conventional concept that biological processes are mostly regulated by genes with protein-coding ability (32). There is much research indicating that lncRNAs participate in the process of tumorigenesis. More recently, lncRNAs are associated with various biological behaviors of PTC, including autophagy (33–35), invasion (36, 37), and metastasis (38) by acting as cancer oncogenes or suppressor genes. Although accumulating evidence has shown that the mRNA expression profile could be regarded as a powerful predictive tool for patients suffering from cancer, the risk stratification based on the lncRNA profile has been shown to have an excellent prognostic value because lncRNAs act in a functional mode that is different from that of mRNA with coding ability (39–42). The abnormal expression of particular lncRNAs in cancer is a response to cancer progression, and they could serve as powerful independent biomarkers for diagnosis and prognosis. However, the prognostic value of lncRNAs in PTC has not been systematically explored. Meanwhile, it is often difficult to accurately risk stratify and make an optimal benefit–harm balance of management of PTC, particularly for the risk stratification of small nodules and prediction of OS.

In this study, we established a risk stratification based on the expression profile of autophagy lncRNAs. In order to identify target lncRNAs, we performed coexpression analysis and obtained 1,283 autophagy-related lncRNAs (|R|>0.3 and P value <0.001). After performing univariate and multivariate analyses, autophagy-related lncRNAs with independent prognostic values were selected to construct a risk score model by using matched expression levels. According to the median risk score, the patients were assigned to the high-risk group and low-risk group. Consistent with our assumptions, patients in the high-risk group tended to have worse OS than that in those in the low-risk group. We draw the ROC curve and calculated its AUC value. All the results showed that the risk stratification could easily classify the risk state of PTC. Among the lncRNAs composing the risk stratification, AC008063.1, AC092279.1, LINC00900, and LINC02454 were protective factors. AC004918.3, AC011297.1, AL162231.2, CRNDE, FAM201A, and TONSL-AS1 were risk-related factors. Besides, we employed the Cox regression analysis and certified that the risk stratification is an independent factor of PTC. At the same time, we evaluated the clinical value of the risk stratification in clinical characteristics including age, gender, tumor, node, metastasis, and TNM staging classification. We also made comprehensive and systematic estimation of tumor-infiltrating cells through different databases and immunosuppressed molecules based on the risk stratification.

The initial intervention of PTC always starts with surgical resection of the gland with primary tumor and the metastatic lymph nodes (43, 44). However, the management of advanced PTC patients with a high risk of recurrent disease and exhibiting radioactive iodine refractoriness is still full of intractability after undergoing total thyroidectomy (45, 46). The patients mentioned above may be candidates to benefit from drug therapy. Therefore, we explored the association between the risk stratification and the efficacy of common chemotherapeutics based on the THCA cohort of TCGA project. The IC50 of sorafenib in the high-risk group is higher than that of the low-risk group. A risk lncRNA CRNDE was chosen for further study, and the experimental evidence showed that knocking down CRNDE might enhance sensitivity to sorafenib in *in vitro* experiments.

We hope the risk stratification based on the autophagy-related lncRNAs helps the clinical worker identify patients with conventionally high risk of PTC and could contribute to the management of patients with PTC.

Our research focused on autophagy-related lncRNAs, and there are certain limitations in our research. First, our studies were based on TCGA cohort and need to be further validated using another additional cohort with a long follow-up time. While low mortality is a characteristic of PTC, there is an absence of cohorts with sufficiently long follow-up. Furthermore, further experiments need to be carried out to determine the mechanisms and functions of these autophagy-related lncRNAs in the tumorigenesis of PTC.

## Data Availability Statement

The original contributions presented in the study are included in the article/**Supplementary Material**. Further inquiries can be directed to the corresponding authors.

## Author Contributions

Conception and design: DZ and RF. Administrative support: KY and YL. Provision of data: YM. Collection and assembly of data: YM. Data analysis and interpretation: YM, FS, and YL. Article writing: YM and ZZ. All authors contributed to the article and approved the submitted version.

## Funding

This study was funded by the National Natural Science Foundation (81702752), Shandong Province Natural Science Foundation (ZR2017BH076), and Shandong Province Science and Technology Key Project (2017GSF18145).

## Conflict of Interest

The authors declare that the research was conducted in the absence of any commercial or financial relationships that could be construed as a potential conflict of interest.

## Publisher’s Note

All claims expressed in this article are solely those of the authors and do not necessarily represent those of their affiliated organizations, or those of the publisher, the editors and the reviewers. Any product that may be evaluated in this article, or claim that may be made by its manufacturer, is not guaranteed or endorsed by the publisher.
