# Supplementary material for: A Comprehensive Risk Assessment and Stratification Model of Papillary Thyroid Carcinoma Based on the Autophagy-Related LncRNAs
Source: Front Oncol. 2022 Feb 24;11:771556. doi: 10.3389/fonc.2021.771556 (PMC8908373; doi:10.3389/fonc.2021.771556)
Supplement: Supplementary file 1 [file DataSheet_1.docx]

# Supplementary Figures


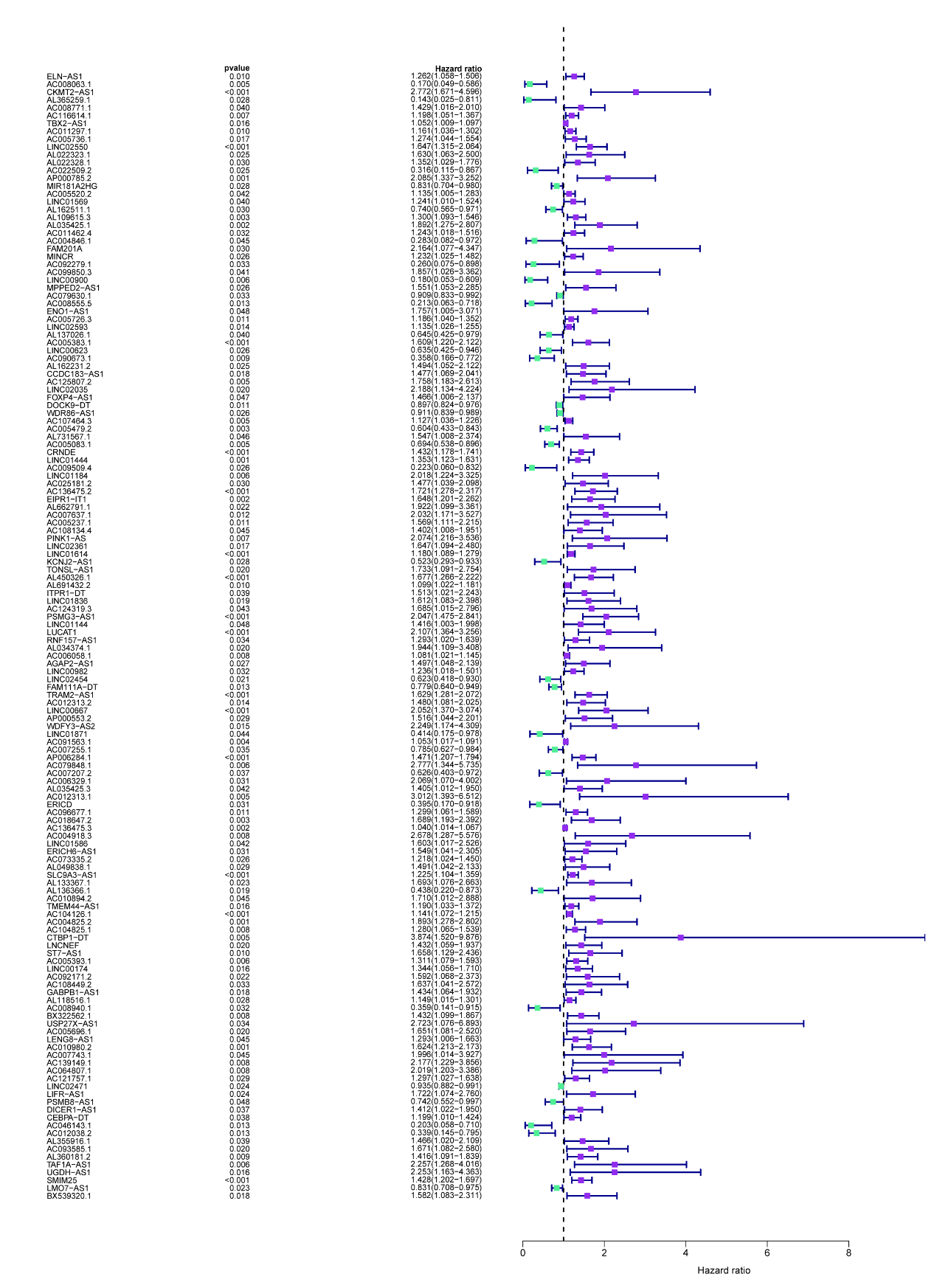


Supplement Figure1

The forest showed the HR (95%CI) and p-value of selected lncRNAs by univariate Cox proportional-hazards analysis.


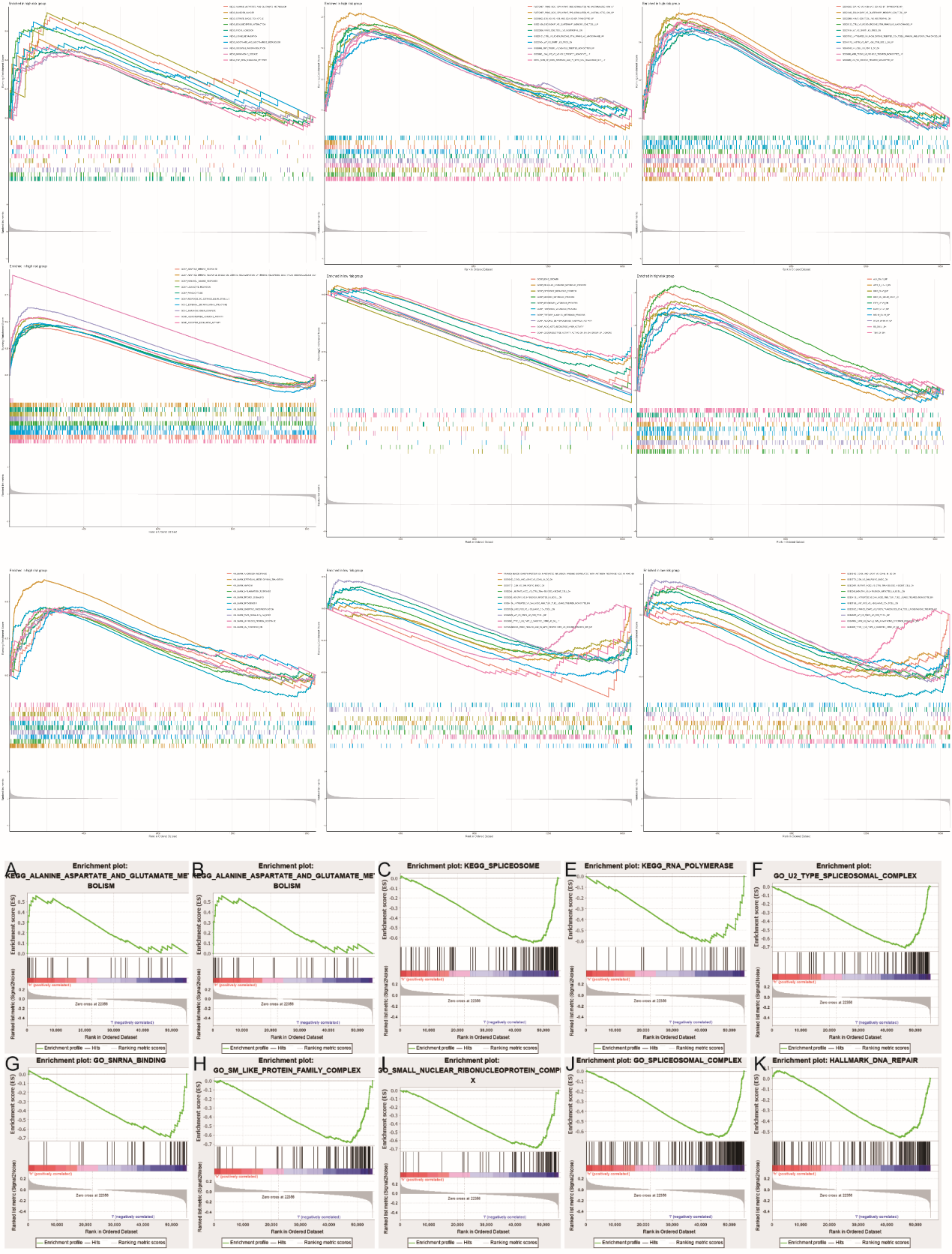


Supplement Figure2

Enriched pathways of the high-risk and low-risk groups based on the risk stratification via GSEA.


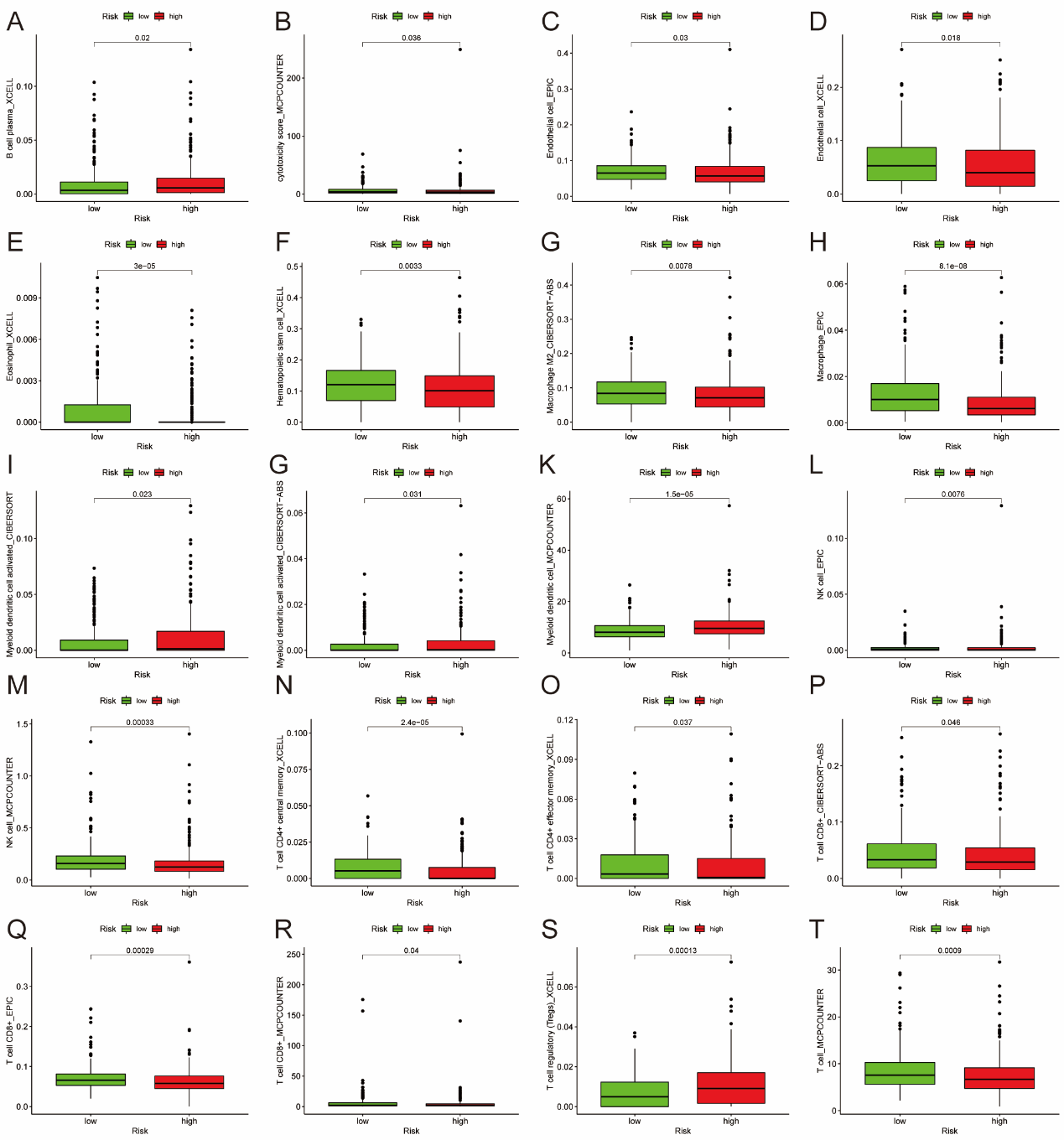


Supplement Figure3

Differences of the immune cells between high risk group and low risk group according to different databases


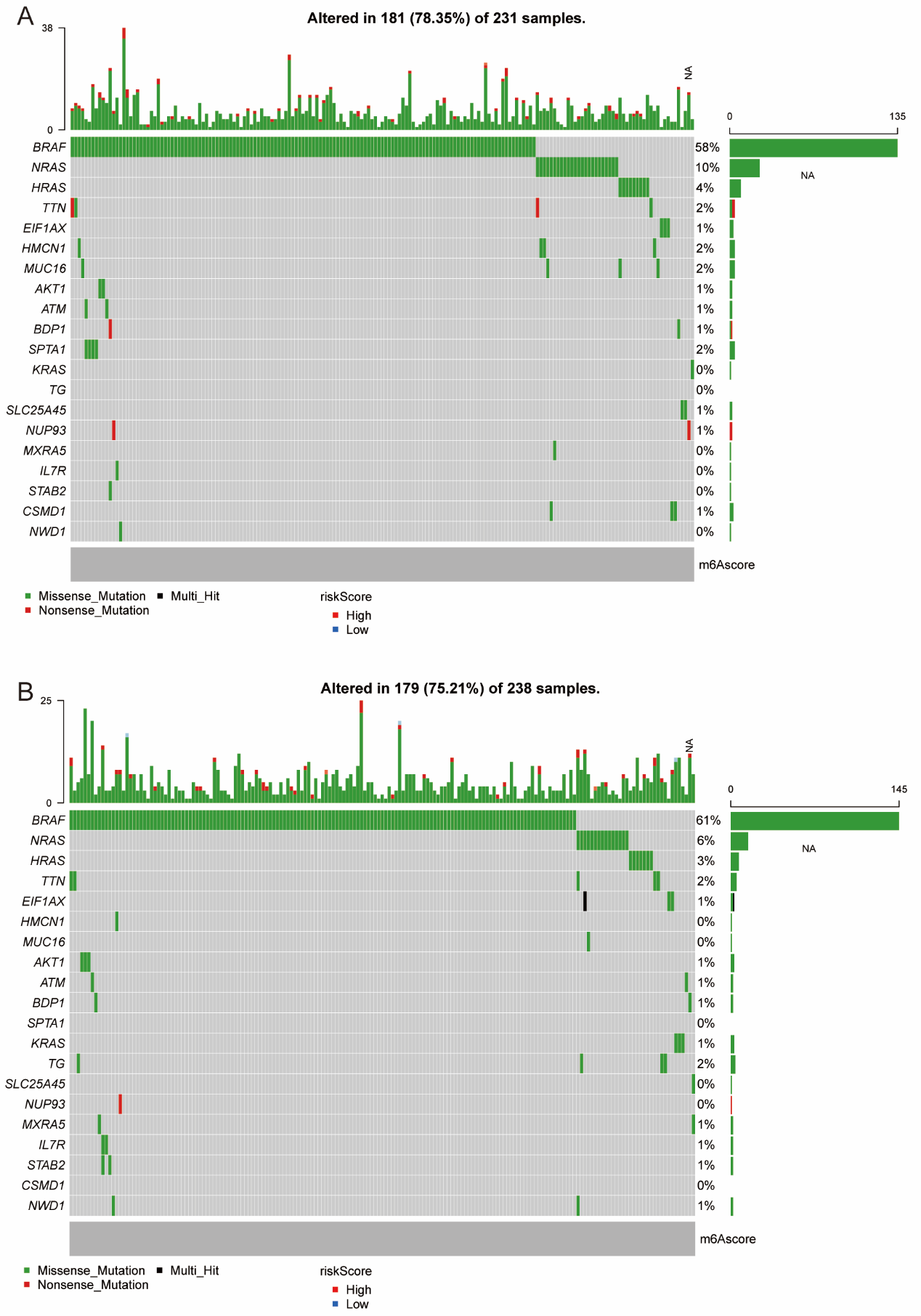


Supplement Figure4

Mutation landscape among the high risk group and low risk group

# Supplementary Tables

Supplement Table1

Primer of CRNDE and ACTB and siRNA sequences of si-CRNDE and si-NC

Supplement Table2

Correlation between the autophagy related lncRNAs and autophagy genes in breast cancer by employing co-expression analysis.

Supplement Table3

Autophagy lncRNAs with prognostic value by univariate Cox regression analysis

HR, Hazard ratio; lncRNA, long noncoding RNA.

Supplement Table4

Multivariate Cox regression results

Supplement Table5

Autophagy gene
