# Supplementary material for: A Comprehensive Risk Assessment and Stratification Model of Papillary Thyroid Carcinoma Based on the Autophagy-Related LncRNAs
Source: Front Oncol. 2022 Feb 24;11:771556. doi: 10.3389/fonc.2021.771556 (PMC8908373; doi:10.3389/fonc.2021.771556)
Supplement: Supplementary file 2 [file Table_1.docx]

| Human CRNDE siRNA sequence from Ribobio company | | |
| --- | --- | --- |
| si‐CRNDE #1 | Sense 5'-3' | GAGAUUCUGAAGAUAAGGATT |
|  | Antisense 5'-3' | UCCUUAUCUUCAGAAUCUCTT |
| si‐CRNDE #2 | Sense 5'-3' | GAUGUGUUUCAAUCUAGAUTT |
|  | Antisense 5'-3' | AUCUAGAUUGAAACACAUCTT |
| si‐CRNDE #3 | Sense 5'-3' | GUUUAUAGACUAUAAUACUTT |
|  | Antisense 5'-3' | AGUAUUAUAGUCUAUAAACTT |
| si-NC | Sense 5'-3' | UUCUCCGAACGUGUCACGUTT |
|  | Antisense 5'-3' | ACGUGACACGUUCGGAGAATT |
| FAM negative control | Sense 5'-3' | UUCUCCGAACGUGUCACGUTT |
|  | Antisense 5'-3' | ACGUGACACGUUCGGAGAATT |
| Sequence of Primers for CRNDE and β-actin | | |
|  | 5’- 3’ | 3’- 5’ |
| CRNDE | CAAGGCTGGTCTGCAAAGTCT | CAACATTTCCAGTGGCATCCTC |
| β-actin | CGCGAGAAGATGACCCAGAT | GGGCATACCCCTCGTAGATG |
