# Supplementary material for: A Comprehensive Risk Assessment and Stratification Model of Papillary Thyroid Carcinoma Based on the Autophagy-Related LncRNAs
Source: Front Oncol. 2022 Feb 24;11:771556. doi: 10.3389/fonc.2021.771556 (PMC8908373; doi:10.3389/fonc.2021.771556)
Supplement: Supplementary file 6 [file Table_5.docx]

| id | coef | HR |
| --- | --- | --- |
| AC008063.1 | -1.74016 | 0.175493 |
| AC011297.1 | 0.171232 | 1.186766 |
| FAM201A | 0.895715 | 2.449087 |
| AC092279.1 | -3.73009 | 0.023991 |
| LINC00900 | -2.38497 | 0.092092 |
| AL162231.2 | 0.671649 | 1.957463 |
| CRNDE | 0.450612 | 1.569273 |
| TONSL-AS1 | -0.79311 | 0.452437 |
| LINC02454 | 0.588168 | 1.800687 |
| AC004918.3 | 2.412627 | 11.16325 |
